# Three human aminoacyl-tRNA synthetases have distinct sub-mitochondrial localizations that are unaffected by disease-associated mutations

**DOI:** 10.1074/jbc.RA118.003400

**Published:** 2018-07-13

**Authors:** Ligia Elena González-Serrano, Loukmane Karim, Florian Pierre, Hagen Schwenzer, Agnès Rötig, Arnold Munnich, Marie Sissler

**Affiliations:** From the ‡ Université de Strasbourg, CNRS, Architecture et Réactivité de l'ARN, UPR9002, F-67084 Strasbourg, France and; the § INSERM UMR 1163, Laboratory of Genetics of Mitochondrial Disorders, Paris Descartes-Sorbonne Paris Cité University, Imagine Institute, F-75015 Paris, France

**Keywords:** aminoacyl tRNA synthetase, mitochondria, translation, neurodegenerative disease, human, dual-localization, leukodystrophy, membrane-anchored, mitochondrial disorder, pontocerebellar hypoplasia

## Abstract

Human mitochondrial aminoacyl-tRNA synthetases (mt-aaRSs) are key enzymes in the mitochondrial protein translation system and catalyze the charging of amino acids on their cognate tRNAs. Mutations in their nuclear genes are associated with pathologies having a broad spectrum of clinical phenotypes, but with no clear molecular mechanism(s). For example, mutations in the nuclear genes encoding mt-AspRS and mt-ArgRS are correlated with the moderate neurodegenerative disorder leukoencephalopathy with brainstem and spinal cord involvement and lactate elevation (LBSL) and with the severe neurodevelopmental disorder pontocerebellar hypoplasia type 6 (PCH6), respectively. Previous studies have shown no or only minor impacts of these mutations on the canonical properties of these enzymes, indicating that the role of the mt-aaRSs in protein synthesis is mostly not affected by these mutations, but their effects on the mitochondrial localizations of aaRSs remain unclear. Here, we demonstrate that three human aaRSs, mt-AspRS, mt-ArgRS, and LysRS, each have a specific sub-mitochondrial distribution, with mt-ArgRS being exclusively localized in the membrane, LysRS exclusively in the soluble fraction, and mt-AspRS being present in both. Chemical treatments revealed that mt-AspRs is anchored in the mitochondrial membrane through electrostatic interactions, whereas mt-ArgRS uses hydrophobic interactions. We also report that novel mutations in mt-AspRS and mt-ArgRS genes from individuals with LBSL and PCH6, respectively, had no significant impact on the mitochondrial localizations of mt-AspRS and mt-ArgRS. The variable sub-mitochondrial locations for these three mt-aaRSs strongly suggest the existence of additional enzyme properties, requiring further investigation to unravel the mechanisms underlying the two neurodegenerative disorders.

## Introduction

Mitochondria are double-membrane organelles with essential activities in cellular energy production as well as in a number of pathways linked to cellular life, disease, aging, and death. They possess their own genome and an independent translation machinery devoted in human to the synthesis of 13 proteins. The latter are hydrophobic subunits of the respiratory chain complexes embedded in the mitochondrial inner membrane (1). The human mitochondrial translation machinery is of dual genetic origin where RNA constituents are encoded by the mitochondrial genome (mt-DNA) and the protein constituents are encoded by the nuclear genome. Previous investigations show that some of the key actors for mitochondrial translation machinery are located at the vicinity of the inner membrane, likely to allow for the direct incorporation of hydrophobic proteins into the membrane (reviewed in Ref. 2). This is for instance the case for the mitoribosome, tethered to the matrix side of the mitochondrial inner membrane (3) via a protuberant domain (the mitochondrial ribosomal protein L45, MRPL45) (4, 5) and for the mitochondrial elongation factor Tu, associated with the inner mitochondrial membrane, independently of the mitoribosome, via a combination of ionic and hydrophobic interactions (6).

Among the proteins involved in mt-DNA expression the aminoacyl-tRNA synthetases (aaRSs)^5^ play a crucial role in mitochondrial protein translation by charging tRNAs with cognate amino acids. In human there is a specific set of aaRSs for mitochondrial localization (mt-aaRSs) distinct from the one for cytosolic localization (with the exceptions of LysRSs and GlyRSs, where cytosolic and mitochondrial versions are encoded by single genes (7)). As for all proteins participating in the mitochondrial translation, the mt-aaRSs are encoded by the nuclear genome and are synthesized within the cytosol, addressed to, and imported into, the mitochondria thanks to mitochondrial targeting sequences (MTS). MTS are cleaved upon entry into the mitochondria (8). Although the macromolecules involved in mammalian mitochondrial translation have been under investigation for many years, there is an increasing interest for the investigation of the human mt-aaRSs since the discovery of a large and growing number of mutations in the encoding genes that are linked to a variety of pathologies (reviewed in Refs. 9–11). Despite being ubiquitously expressed and having a common role in the mitochondrial translation process, mt-aaRSs are impacted in various ways. Their mutations cause an unexpected variety of phenotypic expressions, including mainly neurological disorders but also non-neurological issues. Today, the number of reported cases is steadily growing (12), but the way mutations affect mt-aaRSs in their structure and/or function remains to be elucidated. The fact that comparable mutations in mt-aaRSs lead to diverse diseases, with different ages of onset, and within different tissues represents a confounding issue.

The most prominent case of disease-related mt-aaRS gene concerns *DARS2*, the gene coding for the mitochondrial aspartyl-tRNA synthetase (mt-AspRS). Presently, more than 60 different clinically relevant mutations have been identified and associated with leukoencephalopathy with brainstem and spinal cord involvement and lactate elevation (LBSL) (12, 13). LBSL is a progressive neurodegenerative disorder that affects the brain white matter, and leads mostly to abnormal muscle stiffness and difficulties with coordinating movements. Most affected patients eventually require wheelchair assistance (14). Mutations within *RARS2*, the gene coding for the mitochondrial arginyl-tRNA synthetase (mt-ArgRS), are correlated with pontocerebellar hypoplasia type 6 (PCH6) (15). Symptoms such as severe impairment of brain development, hypotonia, lethargy, poor sucking and/or recurrent apnea appear on the first days after birth. For infants surviving beyond the newborn period, the growth of the head is arrested and progressive microcephaly is observed (16). Up to date, only 22 cases of PCH6 are reported and most of the patients are presently deceased. Investigations performed so far showed no or variable impacts on the canonical properties of the enzymes, indicating that the housekeeping role of the mt-aaRS in the protein synthesis is not the general target of the mutations (17–19).

Here, we investigate the cellular properties of human mt-AspRS and mt-ArgRS, and establish that the two enzymes have different mitochondrial localizations, despite their involvement in the same mitochondrial translation process. In addition, we report new LBSL and PCH6 patients, compound heterozygous for two mutations in *DARS2* and *RARS2*, respectively. We investigate the impact of a series of disease-associated mutations, affecting the mt-AspRS and mt-ArgRS, on newly established cellular properties. Combined with previous work, the present results open new perspectives, which may shed new light on the links between mutations and the related diseases.

## Results

### Mitochondrial aminoacyl-tRNA synthetases have different intra-mitochondrial localizations

**Figure 1.**
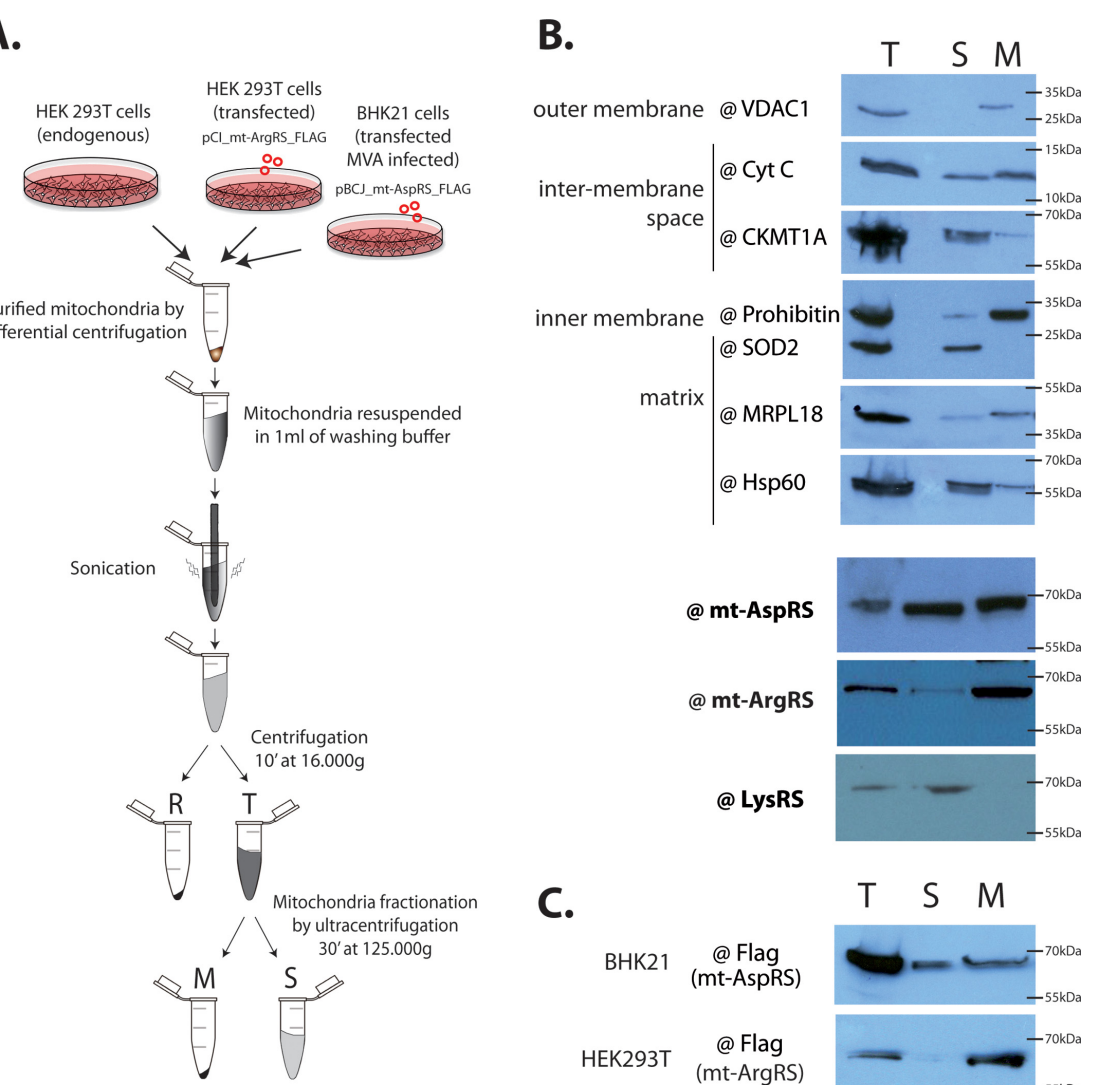
**Sub-mitochondrial localizations of mt-AspRS, mt-ArgRS, and LysRS.** *A,* flow chart of the experimental procedure. Cells are either unmodified HEK293T (for results shown in *B*), or transfected and infected BHK21 for FLAG-tagged mt-AspRS (following the protocol published in Ref. 19) and transfected HEK293T for FLAG-tagged mt-ArgRS (for results shown in *C*). *B,* the purity of the soluble and membrane fractions was assessed by Western blotting detection of mitochondrial marker proteins of known sub-mitochondrial localization: outer membrane (VDAC1), inter-membranes space (Cyt *c* and CKMT1A), inner membrane (prohibitin), and matrix (SOD2, MRPL18 and Hsp60). Mt-AspRS, mt-ArgRS, and LysRS were detected using specific antibodies. *C,* sub-mitochondrial localizations of FLAG-tagged mt-AspRS or mt-ArgRS detected by Western blotting using an antibody against the FLAG tag. **R**, residual fraction; **T**, total mitochondria; **S**, soluble fraction; **M**, membrane fraction.

To establish the sub-mitochondrial localization of mt-AspRS, mt-ArgRS, and LysRS, mitochondria from human embryonic kidney cells (HEK293T) were enriched and fractionated into a soluble fraction (**S** in Fig. 1*A*) containing molecules from the matrix and intermembrane space, and membrane fraction (**M** in Fig. 1*A*) containing molecules from the outer and the inner membranes. Western blots using antibodies against proteins of known sub-mitochondrial localization were used to assess the quality of the fractionation process. The superoxide dismutase 2 (SOD2) (20) is a marker for soluble matrix proteins. The voltage-dependent anion selective channel protein 1 (VDAC1) (21, 22) and prohibitin (23) are integral proteins, anchored to the outer and inner membranes, respectively. The mammalian mt ribosome (3), cytochrome *c* (Cyt c) (24), creatine kinase (CKMT1A) (25), and heat shock protein 60 (Hsp60) (26, 27) are reported to be dual localized in the soluble and membranes fractions as peripheral proteins. All marker proteins were detected in agreement with the literature (Fig. 1*B*), attesting for the quality of the fractionation protocol and the absence of cross-contamination.

The intra-mitochondrial localizations of mt-AspRS, mt-ArgRS, and LysRS were established using specific antibodies. This experiment reveals that the mt-AspRS is distributed in both the soluble and membrane fractions, whereas the mt-ArgRS is exclusively found in the membrane fraction and the LysRS is exclusively found in the soluble fraction (Fig. 1*B*). Identical results, showing different intra-mitochondrial localizations for two of the mt-aaRSs, is observed using anti-FLAG antibodies on BHK21 cells expressing FLAG-tagged mt-AspRS or on HEK293T cells expressing FLAG-tagged mt-ArgRS (Fig. 1*C*).

### Mitochondrial aspartyl-tRNA synthetase and arginyl-tRNA synthetase have different modes of membrane anchoring

**Figure 2.**
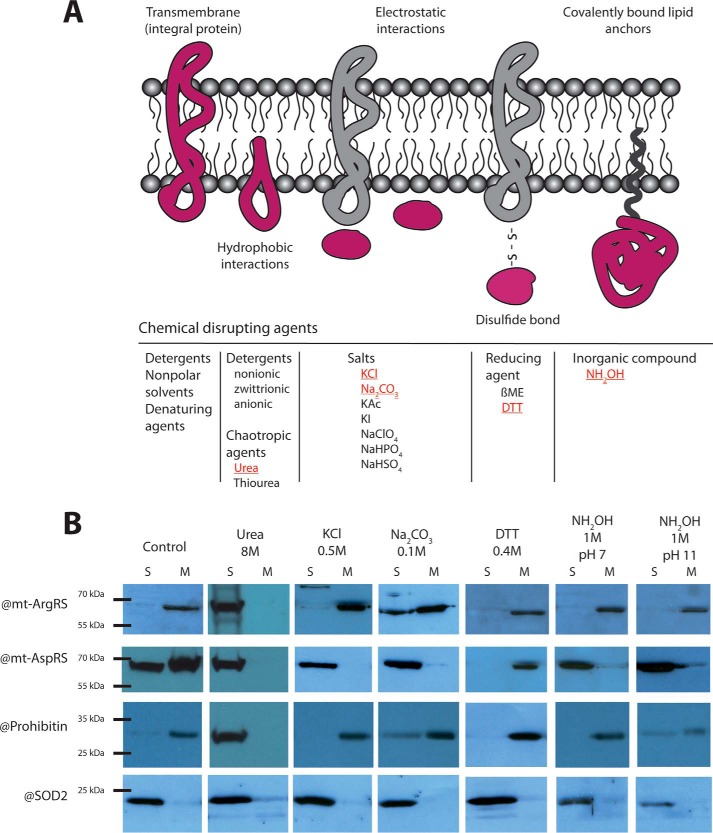
**Different modes of membrane anchoring of mt-AspRS and mt-ArgRS.** *A,* schematic representation of the different modes of protein membrane anchoring or binding. Some chemicals reported to be used for the release of anchored protein are indicated on the scheme, those indicated in *red* have been applied in the present study (for references, see text). *B,* Western blot analysis (using antibodies targeted against mt-ArgRS, mt-AspRS, prohibitin, or SOD2) of the soluble and membranes fractions after treatment as indicated on the *top*.

A protein can either be “integral” and permanently attached to the membrane via a transmembrane domain or hydrophobic region(s); or be “peripheral” and loosely adhered to the membrane through either electrostatic or ionic interactions (via another protein or membrane lipids), disulfide bond interactions, or a covalently bound lipid anchor. A series of disrupting chemical agents have been reported to produce type-specific release of membrane-bound proteins. Examples of chemicals and their specificity of action are provided in Fig. 2*A*. A selection of these disrupting chemical agents (8 m urea (28), 0.5 m KCl (26, 29), 0.1 m Na_2_CO_3_ pH 11 (30–32), 0.4 m DTT (33), 1 m NH_2_OH, pH 7, or 1 m NH_2_OH, pH 11) (34–36) was applied on isolated and enriched mitochondria, prior to the fractionation process. As shown in Fig. 2*B*, all conditions except 0.4 m DTT led to the release of mt-AspRS from the membrane fraction. Of note, in the latter experiment, the portion of mt-AspRS in the soluble fraction is either relocated within the membrane fraction, or lost during the experimental treatment. Conversely, solely urea and carbonate treatments allowed either the total or the partial release of mt-ArgRS, respectively, which remained otherwise anchored to the mitochondrial membrane. SOD2 and prohibitin are detected/analyzed as control experiments from the matrix and the membrane fractions, respectively. Although SOD2 remains soluble whatever is the applied chemical, prohibitin was detected in the soluble fractions upon urea, carbonate, pH 11, and hydroxylamine, pH 11, treatments. Carbonate treatment, pH 11, was reported to lead to the release of some integral proteins with mode of anchoring weaker than the complete transmembrane domain (30–32). Altogether, these results showed that membrane-anchored fractions of mt-AspRS and mt-ArgRS respond to different chemistry, indicating that the mode of anchoring is distinct: salt sensitive for mt-AspRS, indicative of an electrostratic mode of anchoring; salt-resistant but urea-sensitive for mt-ArgRS, indicative of a hydrophobic mode of anchoring for mt-ArgRS.

### Impact of disease-associated mutations on mt-AspRS and mt-ArgRS intra-mitochondrial distributions

The impact of LBSL-related mutations on the solubility of the human mt-AspRS was investigated in a previous study (19). To do so, WT and mutant versions of the mt-AspRS were expressed in modified vaccinia Ankara-infected BHK 21 cells (37). Mt-AspRS being a dimeric enzyme (7) and, as demonstrated here, being dual localized, the choice was made to analyze further mutant mt-AspRSs in the same heterologous expression system. A possible impact of disease-related mutations on the mt-AspRS dual localization will thus be more easily detectable because none of the used antibodies (against either the C-terminal FLAG tag or a peptide specific to the human mt-AspRS) will detect the endogenous hamster mt-AspRS. Mt-ArgRS being a monomeric enzyme and located exclusively in the membrane fraction, any impact on the localization, if observed, would necessarily arise from the disease-related mutation. The choice was thus to express WT and mutant versions of the mt-ArgRS in HEK293T cells (which contains endogenous WT mt-ArgRS). Experiments shown in Fig. 1 confirm that the distribution of both mt-AspRS and mt-ArgRS is conserved in the two types of cultured cells, indicating that the two model systems are appropriate for further investigations.

**Figure 3.**
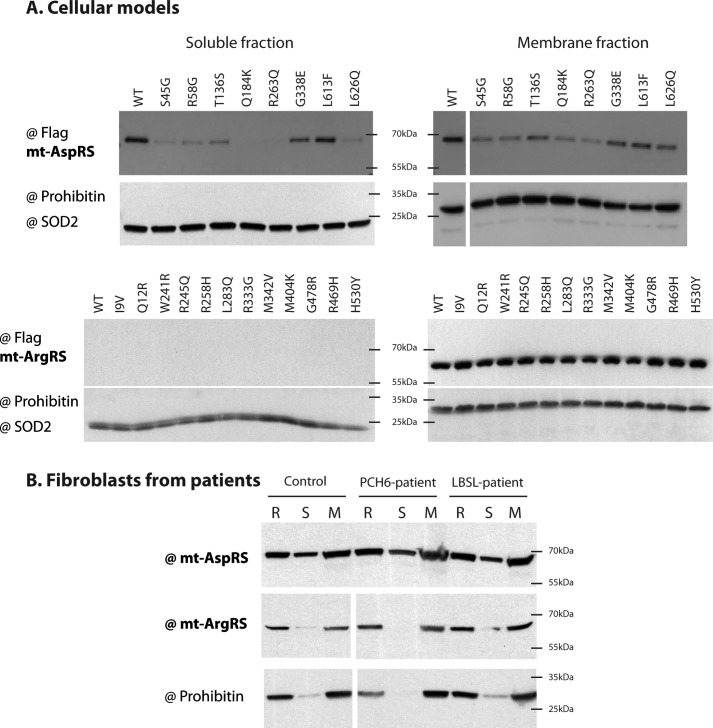
**Impact of disease-associated mutations on the intra-mitochondrial localization.** *A,* cellular models. Shown are representative Western blots of three independent experiments detecting WT and mutant mt-AspRS (*top*) and mt-ArgRS (*bottom*) using anti-FLAG antibody. SOD2 and prohibitin were detected to attest for the quality of the fractionation and used as loading controls for the soluble and membrane fractions, respectively. *B*, fibroblasts from patients. Shown are representative Western blots detecting WT and mutant mt-AspRS, mt-ArgRS, and prohibitin using specific antibodies. *Control* skin fibroblast is from a healthy control person. *PCH6-patient* and *LBSL-patient* correspond to skin fibroblasts from patient 2 (p.T100Cfs*5/p.A10V) and patient 1 (p.R76SfsX5/p.G338E), respectively. **R**, residual fraction; **S**, soluble fraction; **M**, membrane fraction.

The effects of eight LBSL-related and 12 PCH6-related mutations, identified in patients in *DARS2* and *RARS2*, respectively, were investigated regarding their possible impacts on the sub-mitochondrial localization of the corresponding enzyme. WT and mutants mt-AspRS and mt-ArgRS were individually expressed in cellular models and their distributions between soluble (**S**), membranes (**M**), and residual (**R**) fractions were determined by Western blotting. All experiments were repeated three times and the percentage of each fraction was calculated, assuming that **S** + **M** + **R** = 100% of the total expressed protein. Representative Western blots are given in Fig. 3*A* and histograms corresponding to the relative distribution of mt-AspRS variants are given in Fig. S1. None of the PCH6-related mutations alter the expression (Fig. S2) and the membrane-anchored localization (Fig. 3*A*) of the mt-ArgRS. A statistically significant (*p* values < 0.05) reduction in the soluble fraction was observed for Q184K^6^ and R263Q mutants of mt-AspRS (Fig. 3*A*, Fig. S1). Even with variations, no significant effect was noticed in the membranes fractions of mt-AspRS mutants. The residual fraction of the Q184K mutant was significantly increased compared with WT mt-AspRS (*p* value < 0.05), consistent with the lower solubility already observed for this variant (19).

Fibroblasts derived either from the LBSL patient (p.R76SfsX5/p.G338E), from the PCH6 patient (p.T100Cfs*5/p.A10V) (see “Experimental Procedures” for cases reports), or from a healthy control were fractionated following the same procedure. Of note, due to the lower abundance of mitochondria in skin fibroblasts as compared with immortalized HEK or BHK cells, a number 10-fold greater of cells was necessary (∼2 × 10^7^ fibroblast cells as compared with ∼2 × 10^6^ required for HEK or BHK cells per experiment). No difference of growth between healthy and LBSL- or PCH6-derived fibroblasts was noticed. Detections of either the endogenous mt-AspRS or the endogenous mt-ArgRS within the two patient-derived cell lines show distributions strictly comparable with the ones within fibroblasts from healthy control (Fig. 3*B*).

## Discussion

aaRSs are housekeeping enzymes that catalyze the aminoacylation of cognate isoaccepting tRNAs, at least as the main recognized function. The human cytosolic aaRSs have been extensively investigated and the organization of nine of them within a macromolecular complex was established (*e.g.* Refs. 38 and 39). Alternative cellular and/or extracellular activities linked to metabolism, angiogenesis, immune response, inflammation, tumorigenesis, or neuronal development, among other functions, have also been reported (38, 40–42). In contrast, the knowledge regarding cellular organization of mt-aaRSs and possible alternative function(s) remains scarce. It is only recently that a pro-angiogenic function of the rat mt-TrpRS (43) and a cysteine polysulfidation activity of the mice and the human mt-CysRSs (44) have been identified.

In the present study, we establish the sub-mitochondrial localization of three mt-aaRSs and demonstrate that regardless of their common role in the aminoacylation of cognate tRNAs, they have different distributions. The applied fractionation protocol separates soluble proteins from the intermembrane space or the matrix (soluble fraction) from those anchored to the inner or the outer membranes (membrane fraction). We show that the mt-ArgRS is exclusively localized in the membrane, the LysRS is exclusively in the soluble fraction, and the mt-AspRS is dual localized, being present in both soluble and membrane fractions. Multiple localizations have been observed at several instances for mitochondrial proteins. Few reports have in addition established a link between multiple localizations and multiple functions or modulated activities of the corresponding protein (*e.g.* Refs. 29, 45, and 46). Previously, we showed that the human mt-AspRS is processed after mitochondrial importation into three different products of maturation from which two co-exist in the mitochondria (8). This observation open new perspectives in the biological understanding of the mt-AspRS and is considered in line with the discovery of two mature forms of the mt-ribosomal protein of the large subunit MRPL12, generated by a similar multiple steps cleavage process during import and demonstrated to display distinct functions (47). The question now is whether there is a correlation between the two mature forms of mt-AspRS, the two sub-mitochondrial localizations described in the present study, and the possibility of this enzyme to have distinct functions or modulated activities.

We further demonstrate that the mode of membrane-anchoring is different between the membrane fractions of the mt-AspRS and the mt-ArgRS: salt-sensitive for the mt-AspRS, indicating an electrostatic mode of membrane anchoring, and urea-sensitive for the mt-ArgRS, indicating a hydrophobic mode of membrane anchoring. None of the two proteins has a sequence-based predictable transmembrane domain (tested in the TMHMM Server, http://www.cbs.dtu.dk/services/TMHMM-2.0 (48), not shown).^7^ There are several examples where an aaRS is relocated into a membrane upon stimuli. This is either to positively or negatively regulate the translation or to allow the occurrence of an alternate function of the aaRS by removing it from translation. For instance, the human cytosolic LysRS is naturally anchored to the cytosolic multi-synthetase complex but relocated to the plasma membrane upon laminin-dependent phosphorylation. Once at the plasma membrane, the LysRS associates with membrane proteins p67LR and the integrin α6, increasing cellular migration and invasion (49). The authors suggest that the recruitment of a key translational component to regulate cell migration may reduce the level of operational translation machinery (50). As another example, the human cytosolic LeuRS is translocated to the lysosome membrane upon leucine addition. In this situation, LeuRS acts as an intracellular leucine sensor and activates the mammalian target of rapamycin-signaling pathway, regulating translation, cell size, and autophagy (51, 52). Finally, in cyanobacteria, four aaRSs are anchored to the thylakoid membranes thanks to an additional protein domain named CAAD (for cyanobacterial aminoacyl-tRNA synthetases appended domain) (53). The membrane-anchored ValRS was shown to directly interact with the ATP-synthase, linking elements from gene translation and energy production machineries (54). In this case, the aaRS is constitutively anchored to the membrane (and not relocated upon stimuli), a situation similar to what we observed for the human mt-ArgRS.

**Figure 4.**
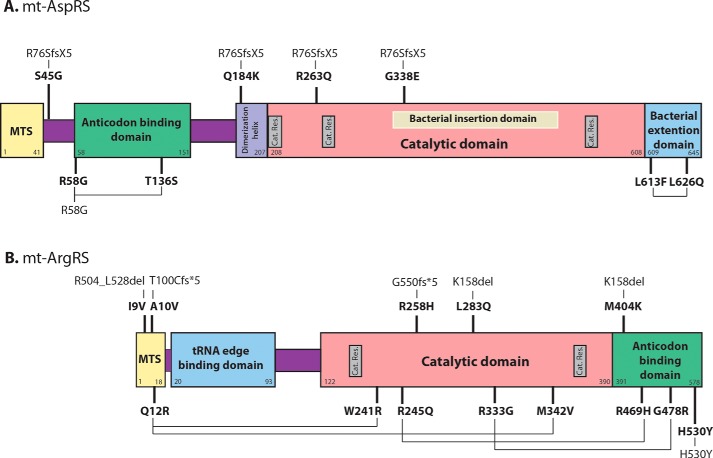
**Investigated disease-associated mutations of human mt-AspRS (*A*) and mt-ArgRS (*B*).** The mt-aaRSs are shown to scale; known functional domains are named and colored. *Cat. Res*. stands for “catalytic residues.” Allelic compositions, as identified in patients, are linked through *black lines*. R76SfsX5 is a truncated variant of mt-AspRS due to the c.228–20_21delTTinsC mutation. T100Cfs*5 and G550fs*5 are truncated variants of mt-ArgRS due to the c.298G_1→A and the c.1651–2A→G mutations, respectively. Mutations investigated in the present study are highlighted in *bold characters*. Rigorously, amino acid conversion of a given mutation should be preceded by the “p” letter to indicate that the protein level is considered. For sake of simplicity, the “p” is omitted. All data are extracted from Ref. 12, which contains all related references. Figures were adapted from Ref. 11.

Over the past 10 years, disease-related mutations affecting *DARS2* and *RARS2* have been reported in patients with LBSL or PCH6 syndromes, respectively (12). Schematic representations of the modular organizations of mt-AspRS and mt-ArgRS are given in Fig. 4. Previous studies, on the same subset of mt-AspRS mutants, revealed that only some had lower aminoacylation activities (mutants L626Q and R263Q) (17, 55). Additional investigations showed that the analyzed mutations have no impact on the mt-AspRS architecture (19), but distinct and variable impacts on mt-AspRS expression (T136S, Q184K, L626Q) (17), dimerization (Q184K) (17), translocation from the cytosol to the mitochondria (S45G) (56), or on *in cellulo* solubility (Q184K, L626Q) (19). Strikingly, mutations R58G and L613F have no noticeable impact on any of these parameters. Regarding PCH6-related mutations of mt-ArgRS, limited investigations have been performed so far. A striking reduction in the amount of the mt-tRNA^Arg^ was found in patient's fibroblasts (with the combination of the K291R mutation and the IVS2 + 5 (A→G) mutation, which causes exon 2 skipping) with, however, the observation that the residual mt-tRNA^Arg^ transcript was almost fully acylated, suggesting that the uncharged mt-tRNA^Arg^ become unstable (15). Lower mt-ArgRS expression and activity have been observed in cultured skin fibroblasts from two patients (with p.R245Q/p.R469H or p.W241R/p.Q12R combinations of mutations). A drastically less significant impact has, however, been reported in a third patient (p.I9V/p.R504_L528del) (18). Altogether, the fact that disease-related mutations impact variably the investigated properties of the proteins is perplexing and points to the fact that the housekeeping role of mt-aaRSs in protein synthesis is not the general target of the mutations, and that different mechanisms are at play in the pathogenesis of mt-aaRS-associated diseases.

In the present study, we have investigated the impact of LBSL- and PCH6-causing mutations on mt-AspRS dual localization and on mt-ArgRS membrane localization, respectively. With the exception of Q184K, none of the investigated mutations significantly impairs the intra-mitochondrial distribution of the corresponding mt-aaRS, neither in cells expressing mutant proteins nor in skin fibroblast cells derived from patients. The Q184K mutation affects the matrix localization of mt-AspRS, consistent with the lower solubility and higher propensity to aggregate, previously determined for this mutant *in vitro* by biophysical approaches (19). It is worth mentioning that the two patient-derived skin fibroblasts are heterozygous compounds and contain a missense mutation and a splicing defect. The splicing defect in the case of a *DARS2*-related LBSL patient was reported to be leaky and to allow the production of a significant amount of WT protein (13, 14). The same authors have further demonstrated the cell-type dependence of splicing of mt-AspRS mRNA and that the mutations have a larger effect on exon 3 exclusion in neuronal cell lines than in non-neural cell lines (57). This may explain the lack of visible impact on skin fibroblast cells expressing both a missense mutation and a splicing defect.

The case of mutant S45G is puzzling. Indeed, it was previously shown that this mutation impairs the import of the protein into isolated mitochondria purified from HEK293T cells, but does not affect the targeting or the processing (cleavage) as demonstrated by *in cellulo* and *in vitro* approaches, respectively (56). In the present study, the S45G mutant is expressed *in cellulo* and shown to be soluble and dually localized in mitochondria similarly to the WT protein. It is thus hypothesized that the S45G mutation, situated near one of the mt-AspRS processing sites (8), may alter the efficiency of the import but not the import itself. This process might then be more sensitive in isolated mitochondria than in a cellular context. Corroborating this hypothesis, the targeting and import of a same protein was demonstrated to be different from one cell or tissue to the other one (58). Also, mutation in the huntingtin protein was shown to affect the mitochondrial import into neurons but not into other cells, leading to premature neuronal death in patients with Huntington disease (59).

### Outlook

We have established the different intra-mitochondrial localizations for the human mt-aaRSs in kidney and skin fibroblasts cells. This distribution should be further investigated in other tissues, *e.g.* in neuronal cells and/or under distinct physiological or pathophysiological contexts. It is worth mentioning that both LBSL and PCH6 are neuronal affections. Similarly, the absence of impact of disease-associated mutations on mt-AspRS or mt-ArgRS intra-mitochondrial localizations does not exclude a possible impact in other cellular contexts. This is consistent with the observation of the “lack of clear biochemical phenotypes (OXPHOS or mitochondrial protein synthesis defects) in skin fibroblasts and myoblasts from most of mutant mt-aaRSs patients,” previously described (60). Similar investigations should now be performed on neuronal cellular models.

The discovery of different localizations and diverse modes of membrane anchoring for two mt-aaRS should be considered in line with the observation that the two related diseases have distinct onsets and degrees of severity. The observation of contrasting scenarios further points to the fact that different mechanisms are likely at play in the pathogenesis of mt-aaRS-associated diseases and suggests distinct intra-mitochondrial roles of the two mt-aaRSs, which will need to be unveiled.

## Experimental procedures

Informed consent for diagnostic and research studies was obtained for all subjects in accordance with the Declaration of Helsinki protocols and approved by local Institutional Review Boards in Paris.

### Cells, biochemical, and chemicals

HEK293T were from Invitrogen. Skin fibroblasts (patient-derived and control) were from the Imagine Institute (Paris). Baby hamster kidney cells strain 21 (BHK21) (ATCC number CRL-12072) and modified vaccinia Ankara strain (MVA-EM24) were gifts from Robert Drillien (IGBMC, Strasbourg). Polyclonal anti-human mt-AspRS was produced in rabbit by the service of antibodies production at the IGBMC (Illkirch) and raised against the peptide ^486^LFLPKEENPREL^497^. Antibodies against human mt-ArgRS, human LysRS, human superoxide dismutase (SOD2), prohibitin, voltage-dependent anion-selective channel (VDAC1), and cytochrome *c* (Cyt c) were purchased from Abcam®. Antibodies against mt creatine kinase (CKMT1A), heat shock protein 60 (Hsp60), and anti-FLAG® were purchased, respectively, from ProteinTech, Bethyl Lab, and Sigma. Mt-ribosomal protein L18 (MRPL18) antibody was a gift from Dr. Entelis (GMGM, Strasbourg). Horseradish peroxidase-conjugated goat anti-rabbit and sheep anti-mouse secondary antibodies were from Bio-Rad and GE Healthcare, respectively. Chemiluminescent detection kit was from Pierce (Thermo Scientific), Mini-Protean® TGX Precast polyacrylamide gels and the Trans-Blot Turbo system were from Bio-Rad. Arrest^TM^ protease inhibitor mixture and polyethylenimine (PEI, linear 25 kDa) were purchased from GBiosciences and Polysciences, respectively. Tryptose phosphate broth (TPB) was from Sigma. Trypsin, penicillin/streptomycin, phosphate-buffered saline (PBS), Dulbecco's modified Eagle's medium, and Glasgow's minimum essential medium (GMEM) were purchased from Gibco. Fetal bovine serum (FBS) was from Eurobio.

### Cases reports

#### 

##### Patient 1

Patient 1, a girl, was born to healthy unrelated parents after a term pregnancy and normal delivery. She did well during her first years of life. At six years, she mentioned short, recurrent and increasingly frequent episodes of tingling sensation of the inferior limbs, originally ascribed to Lyme disease. She subsequently presented recurrent attacks of unilateral lower limb weakness, frequent falls, and gait ataxia and limb intention tremor. Left hemiparesis with pyramidal syndrome, brisk deep tendon reflexes, cerebellar ataxia and *pes cavus* were noted but she could attend school normally. Brain MRI revealed bilateral hyper intensity of pyramidal track, pons, cerebellar peduncles and corpus callosum. Her metabolic work up including plasma lactate and pyruvate was normal. Next-generation sequencing revealed compound heterozygosity for two *DARS2* variants, a missense variant (c.1013G>A; p.G338E) and a previously reported nonsense truncating variant (c.228–21_228–20delinsC; p.R76SfsX5) (13).

##### Patient 2

Patient 2, a girl, was born to second-cousin Turkish parents after a term pregnancy and normal delivery. At day 4, she presented bouts of myoclonic jerks unresponsive to valproate and carbamazepine. She developed myoclonic encephalopathy with trunk hypotonia and inability to follow with eyes at aged 2 months. Brain MRI showed severe sus- and sub-tentorial brain atrophy with pericerebral effusion, with no basal ganglia involvement but NMR spectroscopy evidence of an important lactate peak (cerebrospinal fluid lactate: 4.2 mmol/liter, normal < 2.1). Next-generation sequencing revealed compound heterozygosity for two *RARS2* variants, a missense variant (c.29C>T; p.A10V) and a splicing variant (c.298G-1→A) causing a frameshift and a premature termination codon (p.T100Cfs*5).

### Plasmid constructions for protein expression in mammalian cells

For expression in cultured cells, all sequences were cloned with a downstream FLAG (DYKDDDDK) epitope tag coding sequence, so that all proteins are FLAG-tagged at their C termini. The gene for WT mt-AspRS and those carrying mutations c.172C→G (p.R58G), c.406A→T (p.T136S), c.550C→A (p.Q184K), c.788G→A (p.R263Q), c.1837C→T (p.L613F), and c.1876T→A (p.L626Q) were constructed and cloned as previously described (19). The ones carrying the c.133A>G (p.S45G) and c.1013G>A (p.G338E) mutations were generated by directed mutagenesis on a derivative of pBCJ739.14 (37). The gene for WT mt-ArgRS was cloned into the NdeI and XhoI sites of the pCI vector (Promega) with standard molecular biology procedures. Mutations c.25A→G (p.I9V), c.35A→G (p.Q12R), c.721T→A (p.W241R), c.734G→A (p.R245Q), c.773G→A (p.R258H), c.848T→A (p.L283Q), c.997C→G (p.R333G), c.1024A→G (p.M342V), c.1211T→A (p.M404K), c.1406G→A (p.R469H), c.1432G→A (p.G478R), and c.1588C→T (p.H530Y) were introduced by site-directed mutagenesis using Phusion High-Fidelity DNA Polymerase (Thermo Scientific). The primers used for mutagenesis are listed in Table S1.

### Cell culture and transfection

BHK21 cells were cultured in Glasgow's minimum essential medium supplemented with 10% fetal bovine serum, 1% penicillin/streptomycin, and 5% tryptose phosphate broth in 5% CO_2_ at 37 °C. Transfection of BHK21 cells was performed as described (19). Briefly, cells were washed with PBS, infected with modified vaccinia Ankara virus expressing isopropyl 1-thio-β-d-galactopyranoside-inducible T7 polymerase, and subsequently transfected with plasmid expressing either WT or mutant AspRSs under the dependence of T7 promoter using PEI. Plasmid-PEI complexes were prepared in a ratio of 1:4.5 (w/w) and incubated for 15 min at 20 °C prior to the transfection (5 and 15 μg of DNA per plate of 60 and 150 cm^2^, respectively). Protein expression was induced by addition of 1 mm isopropyl 1-thio-β-d-galactopyranoside.

HEK293T cells and skin fibroblasts were cultured in Dulbecco's modified Eagle's medium supplemented with 10% FBS and 1% penicillin/streptomycin in 5% CO_2_ at 37 °C. HEK293T cells were transfected with PEI (with same procedure and plasmid/PEI ratio as mentioned above) at 50% confluence with constructs expressing mt-ArgRS–FLAG (WT and mutants). The transfected cells were incubated at 37 °C for 3 days and then analyzed by Western blotting.

### Mitochondrial enrichment

Cells were collected, washed with PBS, and resuspended in an isotonic buffer (220 mm mannitol, 70 mm sucrose, 1 mm MgCl_2_, 1 mm EDTA, 10 mm HEPES/KOH, pH 7.4) containing a protease inhibitor mixture. Cells were then disrupted mechanically using 2-mm diameter ceramic beads in a FastPrep-24^TM^ 5G machine (MP Biomedicals). Intact cells, nuclei, and debris (pellet) were discarded after 10 min of centrifugation at 400 × *g* (4 °C). Supernatant was centrifuged 10 min at 12,000 × *g* (4 °C) to collect fraction enriched in mitochondria (∼40 mg were obtained per 150-cm^2^ confluent plate).

### Mitochondrial fractionation

Flow charts of experimental procedures to fractionate mitochondria are given in Fig. 1*A*. Mitochondria (∼30 mg) were resuspended in 1 ml of washing buffer (10 mm K_2_HPO_4_/KH_2_PO_4_, pH 7.5, 300 mm mannitol, 1 mm EDTA, containing a protease inhibitor mixture), and sonicated 6 × 10 s on ice. Sonicated mitochondria were centrifuged during 10 min at 16,000 × *g* (4 °C). The resulted pellet (named residual fraction **R**) corresponds to unbroken mitochondria and aggregates, if any. The supernatant (**T**, for total mitochondria) was further ultracentrifuged 30 min at 125,000 × *g* (4 °C) to separate soluble (**S**) from membranes (**M**) fractions.

### Chemical treatment of mitochondria

Enriched mitochondria (∼30 mg/experiment) were sonicated 6 × 10 s on ice in the presence of different chemical agents (1-8 m urea, 0.1 m Na_2_CO_3_, pH 11, 0.5 m KCl, 0.4 m DTT, or 1 m NH_2_OH, at pH 7 and 11) in 1 ml of 10 mm K_2_HPO_4_/KH_2_PO_4_, pH 7.5, 300 mm mannitol, 1 mm EDTA, and containing a protease inhibitor mixture for 10 min at 20 °C. After sonication, mitochondria underwent the fractionation protocol as described above.

### Western blotting

Soluble (**S**) fractions were concentrated up to a volume of 80 μl using Amicon → Ultra-0.5 centrifugal filters (10K, Millipore). Residual (**R**) and membrane (**M**) fractions were solubilized in 80 μl of washing buffer. The protein concentration of each fraction was quantified using Bradford assay. All fractions were supplemented with 20 μl of Laemmli dissociating buffer, heated at 95 °C for 10 min. Twenty μl of each fraction (containing ∼20 μg of proteins for total, ∼140 μg for the soluble fraction, and ∼65 μg for the membrane fraction) were loaded on a 10% SDS-PAGE. Proteins were blotted on a polyvinylidene difluoride membrane and detected with specific antibodies. Chemiluminescent detection was carried out using the Pierce Detection Kit according to the manufacturer's instructions.

Autoradiographs were digitized using Epson Perfection 3490 Photo. For comparison between mt-aaRS WT and mutants, the relative amount of proteins was estimated from band intensities using ImageJ software (61), and corrected based on SOD2 and prohibitin intensities as loading controls. Soluble membranes and residual fractions obtained from each set of experiments have been quantified out of a same autoradiograph, assuming that all mt-aaRS protein has been recovered during treatment and percentage of each fraction in individual experiments has been calculated (**R** + **S** + **M** = 100%) so that the relative distribution of proteins in each fraction for each condition is independent of the expression rate. Mean values and S.D. were calculated out of three independent replicates. Values were then normalized for comparison purposes to the corresponding WT fraction, artificially set to a value of 1. Due to the low abundance of mt-AspRS mRNA when compared with other mt-aaRS mRNAs in skin cells and when compared with other cells (http://biogps.org),^7^ the detection of the corresponding protein by Western blotting experiments was harder to reproduce in the three fibroblast cell lines.

## Author contributions

L. E. G.-S., L. K., F. P., H. S., A. R., A. M., and M. S. validation; L. E. G.-S., L. K., and M. S. writing-original draft; M. S. supervision; M. S. funding acquisition; L. E. G.-S. and L. K. performed experiments; F. P. cultivated fibroblasts from patients and healthy control; H. S. contributed to the early stages of the work; A. R. and A. M. diagnosed LBSL and PHC6-related patients and provided clinical data about patients.

## Supplementary Material

Supporting Information

## Footnotes

6 Rigorously, amino acid conversion of a given mutation should be preceded by the “p” letter to indicate that the protein level is considered and the amino acid change should be indicated using the three-letter nomenclature. For sake of simplicity, the “p” is omitted and the one-letter code is used so that the mutation p.Gln184Lys is named here Q184K.

7 Please note that the JBC is not responsible for the long-term archiving and maintenance of this site or any other third party hosted site.

## Footnotes

5 The abbreviations used are:

mt-aaRS: mitochondrial aminoacyl-tRNA synthetase
LBSL: leukoencephalopathy with brainstem and spinal cord involvement and lactate elevation
HEK: human embryonic kidney
mt-ArgRS: mitochondrial arginyl-tRNA synthetase
SOD2: superoxide dismutase 2
VDAC1: voltage-dependent anion selective channel protein 1
PCH6: pontocerebellar hypoplasia type 6
PEI: polyethylenimine
BHK: baby hamster kidney.
