# Supplementary material for: Three human aminoacyl-tRNA synthetases have distinct sub-mitochondrial localizations that are unaffected by disease-associated mutations
Source: J Biol Chem. 2018 Jul 13;293(35):13604–15. doi: 10.1074/jbc.RA118.003400 (PMC6120215; doi:10.1074/jbc.RA118.003400)
Supplement: Supporting Information [file supp_293_35_13604__index.html]

Three human aminoacyl-tRNA synthetases have distinct sub-mitochondrial localizations that are unaffected by disease-associated mutations — Intra-mitochondrial distributions of mt-AspRS and mt-ArgRS — Three human aminoacyl-tRNA synthetases have distinct sub-mitochondrial localizations that are unaffected by disease-associated mutations — Intra-mitochondrial distributions of mt-AspRS and mt-ArgRS — Supporting Information 

# Three human aminoacyl-tRNA synthetases have distinct sub-mitochondrial localizations that are unaffected by disease-associated mutations

## Supporting Information

- Supplementary material - two supplementary figures and one table
