# Supplementary material for: Three human aminoacyl-tRNA synthetases have distinct sub-mitochondrial localizations that are unaffected by disease-associated mutations
Source: J Biol Chem. 2018 Jul 13;293(35):13604–15. doi: 10.1074/jbc.RA118.003400 (PMC6120215; doi:10.1074/jbc.RA118.003400)
Supplement: Supporting Information [file supp_RA118.003400_137357_3_supp_167537_pbqzjs.docx]

**SUPPLEMENTARY MATERIAL**

**Three human aminoacyl-tRNA synthetases have distinct sub-mitochondrial localizations that are unaffected by disease-associated mutations**

**Ligia Elena GONZALEZ-SERRANO^1,‡^, Loukmane KARIM^1,2,‡^, Florian PIERRE^1^, Hagen SCHWENZER^1,3^, Agnès RÖTIG^4^, Arnold MUNNICH^4^ and Marie SISSLER^1,^***

From the ^1^Université de Strasbourg, CNRS, Architecture et Réactivité de l’ARN, UPR9002, F-67000 Strasbourg, France; ^2^Present address: Center for Inflammation, Translational and Clinical Lung Research (CILR), Lewis Katz School of medicine, Temple University - Philadelphia, PA 19140 USA; ^3^Present address: Department of Oncology, University of Oxford, Oxford, United Kingdom; ^4^INSERM UMR 1163, Laboratory of Genetics of Mitochondrial Disorders, Paris Descartes - Sorbonne Paris Cité University, Imagine Institute, Paris, France

**Running title:** Intra-mitochondrial distributions of mt-AspRS and mt-ArgRS

**^‡^**These authors contributed equally to this work and should be considered as co-first authors. Their names are listed alphabetically.

*To whom correspondence should be addressed:

Marie Sissler, IBMC - 15 rue René Descartes, 67084 Strasbourg Cedex – France, Phone: +33 (0)3 88 41 70 62; Fax: +33 (0)3 88 60 22 18; E-mail: [m.sissler@ibmc-cnrs.unistra.fr](mailto:m.sissler@ibmc-cnrs.unistra.fr)

**Keywords:** dual localization, membrane-anchored, neurodegenerative disease, aminoacyl-tRNA synthetase, human mitochondria, mutation, pontocerebellar hypoplasia, leukodystrophy, mitochondrial disorder


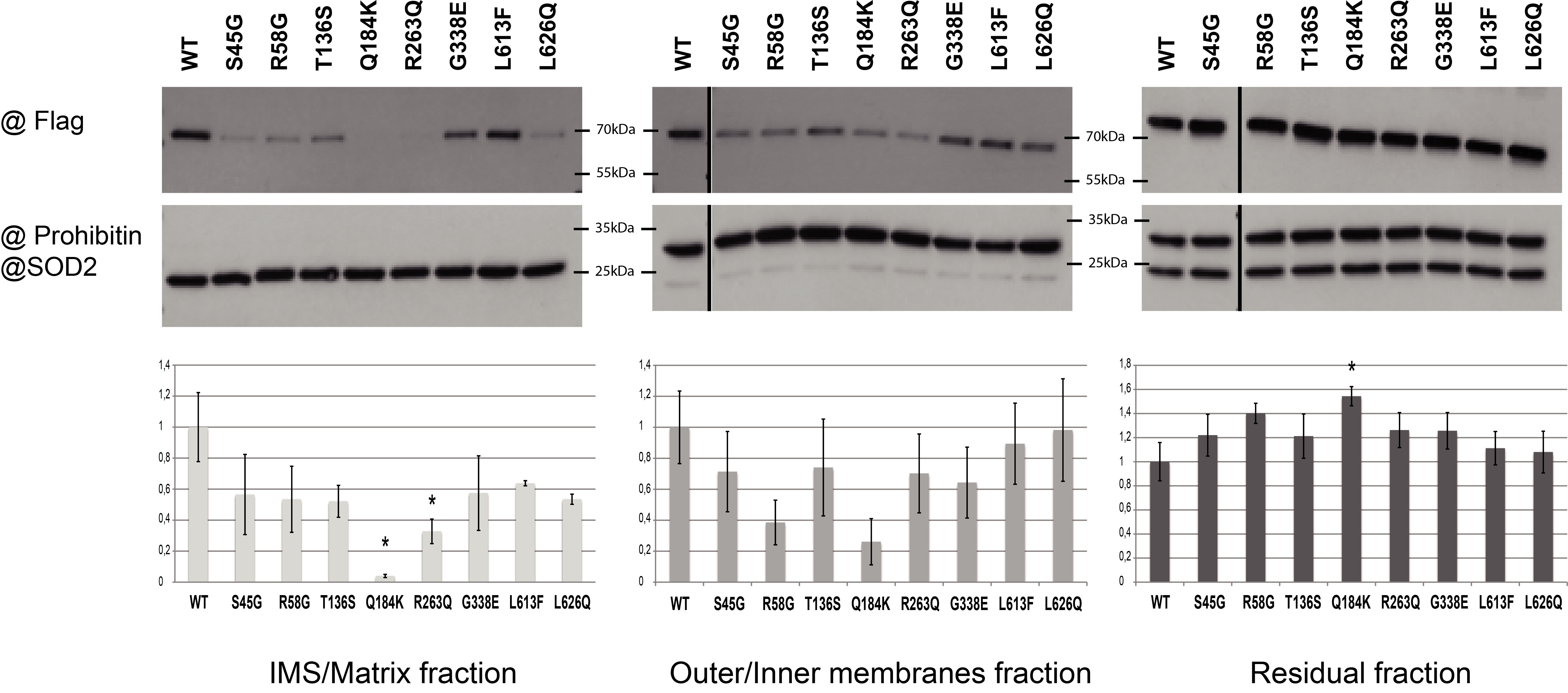


**Supplementary Figure 1: impact of LBSL-related mutations on mt-AspRS distribution in mitochondrial sub-fractions.** Relative amounts of polypeptides were estimated, normalized according to the WT mt-AspRS and arbitrarily set to a value of 1 in all fractions. Errors bars illustrate the standard deviations calculated from the three sets of independent experiments. *p < 0.05 based on Student’s *t*-test.


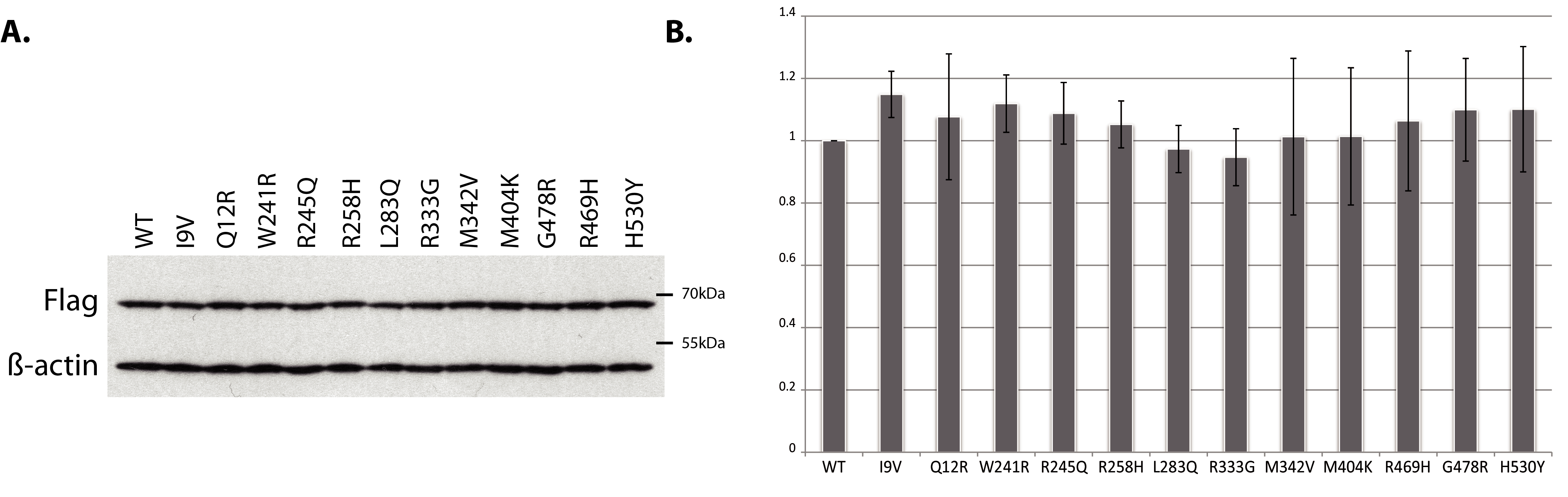


**Supplementary Figure 2: impact of PCH6-related mutations on mt-ArgRS expression. A.** Representative western blots of three independent experiments detecting with anti-Flag antibodies the WT and mutants mt-ArgRS expressed in HEK293T cells. Expression of the variants was normalized using ß-actin (40 kDa) as a loading control. **B.** The relative amounts of polypeptides were estimated, normalized according to the WT mt-ArgRS and arbitrarily set to a value of 1. Errors bars illustrate the standard deviations calculated from the three sets of independent experiments. *p < 0.05 based

**Table S1: List and sequence of the primers used for mutagenesis**

Affected nucleotides are shown in bold and underlined

| **Mutation** | **Name** | **Sequence 5’ → 3’** |
| --- | --- | --- |
| c.25A>G | p.I9V-Forward | GCTTTCGCCGCGCT**G**TTGCTTGCCAGC |
|  | p.I9V-Reverse | GCTGGCAAGCAA**C**AGCGCGGCGAAAGC |
| c.35A>G | p.Q12R-Forward | GCTATTGCTTGCC**G**GCTTTCCAGAGTG |
|  | p.Q12R-Reverse | CACTCTGGAAAGC**C**GGCAAGCAATAGC |
| c.721T>A | p.W241R-Forward | GCACTTTCACTG**A**GGCAAAAATTTCG |
|  | p.W241R-Reverse | CGAAATTTTTGCC**T**CAGTGAAAGTGC |
| c.734G>A | p.R245Q-Forward | CACTGTGGCAAAAATTTC**A**GGACTTGAGCATTGAAG |
|  | p.R245Q-Reverse | CTTCAATGCTCAAGTCC**T**GAAATTTTTGCCACAGTG |
| c.773G>A | p.R258H-Forward | CATTCGGGTTTACAAGC**A**TCTGGGAGTATATTTTG |
|  | p.R258H-Reverse | CAAAATATACTCCCAGA**T**GCTTGTAAACCCGAATG |
| c.848T>A | p.L283Q-Forward | GGTCTTAAAGTTGC**A**GGAGAGTAAAGGAC |
|  | p.L283Q-Reverse | GTCCTTTACTCTCC**T**GCAACTTTAAGACC |
| c.997C>G | p.R333G-Forward | GCTGCTATAGAT**G**GAATGGACAAG |
|  | p.R333G-Reverse | CTTGTCCATTC**C**ATCTATAGCAGC |
| c.1024A>G | p.M342V-Forward | GGACAAGTATAATTTTGATACA**G**TGATATATGTGACAGATAAAGG |
|  | p.M342V-Reverse | CCTTTATCTGTCACATATATCA**C**TGTATCAAAATTATACTTGTCC |
| c.1211T>A | p.M404K-Forward | GAGATTCAATTAAGGA**A**GCTACAGAACATGGC |
|  | p.M404K-Reverse | GCCATGTTCTGTAGC**T**TCCTTAATTGAATCTC |
| c.1406G>A | p.R469H-Forward | CACACACGCCC**A**CCTCCACAGTTTG |
|  | p.R469H-Reverse | CAAACTGTGGAGG**T**GGGCGTGTGTG |
| c.1432G>A | p.G478R-Forward | GGAAGAGACTTTT**A**GATGTGGGTACC |
|  | p.G478R-Reverse | GGTACCCACATC**T**AAAAGTCTCTTCC |
| c.1588C>T | pH530Y-Forward | CCTTCTAACTTTAAGT**T**ATCTTGCAGCTGTGG |
|  | pH530Y-Reverse | CCACAGCTGCAAGAT**A**ACTTAAAGTTAGAAGG |
